# The long-term and short-term effects of interest rate volatility on corporate bankruptcy risk: An industry and supply chain perspective

**DOI:** 10.1371/journal.pone.0317185

**Published:** 2025-02-07

**Authors:** Lingfei Chen, Kai Zhang, Xueying Yang

**Affiliations:** 1 High School of Economics and Business, Al-Farabi Kazakh National University, Almaty, Republic of Kazakhstan; 2 School of Business, Ningbo University, Ningbo, China; Gabriele d’Annunzio University of Chieti and Pescara: Universita degli Studi Gabriele d’Annunzio Chieti Pescara, ITALY

## Abstract

While higher interest rates increase the cost of credit financing for businesses, this study finds that the direct impact of this traditional credit transmission mechanism on corporate bankruptcy risk is limited. Instead, our research reveals that changes in corporate behavior induced by rising debt financing costs are the root cause of bankruptcy risk. In the short term, an increase in interest rates drives businesses to substitute supply chain financing for credit financing in pursuit of profit maximization. This mismatch of short-term debt and long-term investments undermines the sustainability of the supply chain, ultimately reducing financial security—sacrificing safety for profitability. In the long term, higher interest rates exacerbate the overcapacity problem in industries, increasing the unsustainability of the production and sales balance. Using data from China’s construction industry, this study empirically tests these findings and, based on the main conclusions, provides policy suggestions regarding the long- and short-term effects of monetary policy on the sustainable development of China’s construction industry: (1) focus on short-term interest rate risks and be vigilant against commercial credit bubbles; (2) long-term monetary policy should prioritize industrial structure optimization.

## 1. Introduction

Governments often use interest rates as an essential market price to regulate the market economy. Interest rate adjustments significantly impact society and the economy, from the industrial structure [1] and macro-economy to individual consumer habits and social culture. The regulatory ability and speed of interest rates are closely related to the level of marketization in societal development. In particular, interest rate fluctuations have varied impacts on enterprises across industries and at different stages of economic marketization [2, 3]. Capital-intensive industries tend to provide the quickest feedback on changes in market interest rates and can provide the most informative monetary policy effects [2]. This study uses the data from China’s pillar industry, the construction industry, to study how the fluctuation of domestic market interest rates affects the bankruptcy risk of capital-intensive industries in China.

Based on relevant literature, the economic impact of interest rates on enterprises or industries primarily focuses on the following three aspects. The first aspect is the credit transmission mechanism. According to the traditional credit transmission mechanism of monetary policy, the impact of interest rates on enterprises is mainly through the cost of debt financing [4, 5]. However, the level of marketization of the economic system significantly affects the transmission effect. Qian Xuesong [6] (2008) [6] argued that the monopoly bank structure and underdeveloped corporate bond market affect the credit transmission of China’s monetary policy. Similarly, Zhou et al. (2022) [7] found that the severe impact of the COVID-19 pandemic has disrupted the steady state of the financial system, and the lag in monetary policy would be prolonged. The second aspect is the financing structure. Besides the traditional credit transmission mechanism, many scholars have found that the adjustment of interest rates can also change the financing structure of enterprises, as enterprises may use commercial credit to replace bank loans to cope with the credit tightening caused by interest rate increases [8–10]. Yadav AKK and Panda AK (2024) [2] demonstrated that trade credit plays a significant role in monetary policy transmission. Private enterprises with higher financing costs in China tend to be more sensitive to interest rates than state-owned enterprises [11]. Consequently, the substitution effect of interest rates on this financing channel is more significant for non-state-owned enterprises [12, 13]. The third aspect is capital reallocation. The change in interest rates will lead to the reallocation of capital among industries with different profit margins [14, 15]. Additionally, different industries have different sensitivities to unified monetary policy regulation [16], and low interest rates facilitate the flow of capital to more efficient industries. Similarly, Baqaee et al. (2024) [17] found that low interest rates facilitate the reallocation of capital to profitable firms, thereby alleviating cross-sectional mismatch issues. Lippi [18] (2005) [18] believed that high-interest-rate policies are beneficial to promoting the survival of the fittest in industries. Moreover, several studies have shown [19–21] that Chinese enterprises are gradually displaying the characteristics of “unrealized profits” and “financialization”, with their operating conditions increasingly influenced by market interest rates.

In the literature, the definition of the construction industry is frequently traced back to the International Standard Industrial Classification(ISIC, 1968). Historically, the industry’s scope mainly encompassed the construction of buildings and civil engineering projects, including new construction, renovation, repair, demolition, and related assembly works. Subsequently, the definition of the construction industry was broadened to include supporting service industries, such as consulting and design [22]. From an industrial chain perspective, the construction industry is currently divided into primarily three sub-industries: the building materials industry, the construction industry (including construction and installation), and the construction services industry (mainly represented by the real estate sector) [23, 24]. Compared to other industries, the construction industry not only consumes a significant amount of energy (accounting for one-third of the world’s energy consumption [25]) but also wields considerable economic influence (representing an average of 10% of the global gross domestic product [26], and even higher in developed economies [27]), making it a crucial player in global development. Furthermore, the construction industry is characterized by immobility, durability, complexity, and high costs [28, 29]. Therefore, bankruptcies in the construction industry have a profound impact on the economy. When examining the reasons for bankruptcy, the focus often lies on internal factors, such as an imbalance between debt and profitability [30]. In terms of research methods, most studies on corporate bankruptcy risk rely on low-frequency financial data and typically use methods such as Altman’s Z-score [31], neural networks [32, 33], and machine learning techniques like AdaBoost [34]. The relevant literature has conducted extensive research on how interest rates affect the development of enterprises and industries, providing this study with solid economic theories and empirical facts. Building on this foundation, this study expands upon the following unique perspectives: First, this study investigates interest rate risk from the perspective of corporate bankruptcy. Previous studies have focused on the operating performance and structural adjustment of enterprises under interest rate policies. However, limited research has examined interest rate risk from the perspective of corporate bankruptcy. Second, starting from the traditional credit transmission mechanism of monetary policy, this study further expands the research on credit transmission theory in default risk from the perspectives of financing structure and the balance of production and sales. Third, in terms of application, this study examines the bankruptcy risk of the entire construction industry chain from the construction industry’s perspective. As an important pillar industry of the economy, the majority of existing literature primarily focuses on the risks of the real estate industry and cannot comprehensively capture the industrial risks of interest rates. To address the above issues, the remaining content of this study is structured as follows: Section 2 applies an improved contingent claims analysis method to analyze corporate bankruptcy risk using the construction industry as a sample. Section 3 examines the impact of interest rate fluctuations on the risk of the construction industry and its transmission mechanisms (short-term and long-term effects of interest rate fluctuations, respectively).

## 2. Risk of corporate bankruptcy

### 2.1 Principles of contingent claims analysis

This study employs the Contingent Claims Analysis (CCA) method to calculate the firms’ default risk. Contingent claims refer to the right of future earnings depending on the value of another asset. Financial derivatives such as options are typical contingent claims. Merton (1973) [35] conceptualized the firm’s equity and expected debt loss as call options and put options, respectively, with the market value of firm assets serving as the underlying asset and the debt acting as the exercise price. He utilized the Black-Sholes Model (BSM) of option pricing developed by Black Fischer and Myron Scholes (1973) [36] to establish the theoretical framework of the CCA method. This groundbreaking approach enabled the analysis of corporate default risk through the Black-Scholes option pricing formula. The most notable application of the CCA method is the KMV model proposed by Crosbie and Bohn [37] (2002) [37]. The KMV model applies the CCA method to estimate the Default Distance of the firm, using historical default data to simulate its probability distribution and then transforming the estimated the Default Distance into the expected default probability (EDF) via a smooth the Default Distance function.

According to the conclusion of the BSM, the price of a European call option is given by:

$$C_{t}=S_{t}N(d_{1t})-K_{t}e^{-rT}N(d_{2t})$$ (1)

Where:

$$d_{1t}=\frac{ln{(S}_{t}/K_{t})+(r_{t}+{\sigma_{t}}^{2}/2)(T-t)}{\sigma_{t}\sqrt{\left(T-t\right)}}$$ (2)

$$d_{2t}=\frac{ln{(S}_{t}/K_{t})+(r_{t}-{\sigma_{t}}^{2}/2)(T-t)}{\sigma_{t}\sqrt{\left(T-t\right)}}=d_{1t}-\sigma_{t}\sqrt{\left(T-t\right)}.$$ (3)

Here, N(·) represents the cumulative standard normal distribution.

According to the theory of CCA, the market value of a listed company(*C*_*t*_) can be directly obtained, while the market value of its assets(*S*_*t*_) is the variable to be determined. The enterprise’s due debt(*K*_*t*_) is typically represented as the sum of current liabilities and half of non-current liabilities. The time interval(*T*) corresponds to the actual interval between consecutive financial reports, typically based on the timing of the four quarterly reports. The risk-free asset yield(*r*_*t*_) is represented by the yield to maturity of China’s one-year Treasury bonds. For the volatility of asset market value(*σ*_*t*_), the method of Bensoussan A et al. [38] is utilized, which derives *σ*_*t*_ based on the corresponding market value volatility(*σ*_*Et*_):

$$\sigma_{t}=\frac{C_{t}}{N(d_{1t})S_{t}}\sigma_{Et}$$ (4)

Where *d*_1*t*_ is defined in Eq (2).

The variables *S*_*t*_ and *σ*_*t*_ are obtained by solving the above four equations. Subsequently, the firm’s bankruptcy risk index, also known as the Default Distance(DD), is calculated as:

$${DD}_{t}=\frac{S_{t}}{K_{t}}$$ (5)

The Default Distance is the ratio of the market value of a firm’s assets to its liabilities, indicating the level of asset coverage for debt obligations. When the value is less than 1, the firm is considered insolvent. Therefore, a higher Default Distance corresponds to a lower bankruptcy risk.

The probability of default, another output of CCA, refers to the likelihood that the asset’s market value that the market value of a firm’s assets falls below the default threshold: Prob(*S*_*t*_<*K*_*t*_). Given that the distribution of stock prices is known, the default probability can be obtained from the framework of CCA, deduced as follows:

The logarithm of asset market value, derived from Ito’s lemma(Detailed derivation process can refer to: John Hull, options futures and other derivatives, eighth edition, China Machine Press, pp. 243–244.), follows the process:

$$lnS_{t}∼N[lnS_{0}+(r_{t}-{\sigma_{t}}^{2}/2)(T-t),{\sigma_{t}}^{2}(T-t)]$$ (6)

Where *S*_0_ is the initial market value of the asset. Thereby,

$$lnS_{t}-lnK_{t}\sim N[ln(S_{0}/K_{t})+(r_{t}-{\sigma_{t}}^{2}/2)(T-t),{\sigma_{t}}^{2}(T-t)]$$ (7)

Launch the probability of default under asset price is log-normal:

$$Prob(S_{t}<K_{t})=Prob(lnS_{t}<{lnK}_{t})=N\left(-\frac{ln(S_{0}/K_{t})+(r_{t}-{\sigma_{t}}^{2}/2)(T-t)}{\sigma_{t}\sigma \sqrt{T-t}}\right)$$ (8)

It can be demonstrated that the Default Distance and the probability of default are consistent indicators for measuring the bankruptcy risk of a firm. Therefore, this study primarily uses the Default Distance to evaluate enterprise bankruptcy risk.

The CCA method comprehensively provides a comprehensive reflection of both financial data, which captures historical business conditions, and stock price data, which incorporates the latest market information. Unlike low-frequency variables based on historical financial data (such as debt-to-asset ratio, current ratio, and Z-Score [39], the Default Distance can integrate more timely market information with a higher frequency. In contrast to risk indicators solely based on market data (such as Value at Risk, VaR), the Default Distance can partially correct for the misinterpretation of risk caused by irrational market sentiment. However, practical research applications of this method still face certain challenges, particularly in terms of methodological implementation and market efficiency. The main challenges are as follows.

First, there is the issue of matching mixed-frequency data. For the same company across different reporting periods, its stock is treated as a completely separate call option. In other words, these call options differentiate option cycles based on financial report publication points. Most literature selects the last trading day of a specific financial reporting period for sampling market value data. While stock price fluctuations are seen as updates on important information related to the listed company, financial statements, as critical information on the operating condition of a listed company, will undoubtedly cause fluctuations in stock prices upon disclosure. Therefore, the timing of selecting stock market value is crucial when using the CCA method to calculate corporate default risk. Second, there is the issue of irrational volatility. Since the CCA method relies on stock price data, and stock prices often exhibit irrational fluctuations, this can temporarily lead to overestimated or underestimated default risk calculations. Third, there is the problem of financial fraud. Given that the CCA method depends on information from financial reports, financial misreporting can distort critical data, such as corporate debt, thus diminishing the accuracy of the default risk assessments produced by the CCA method.

Given these challenges, this study aims to mitigate the three aforementioned issues in sample and data processing by adhering to institutional practices and conventions for financial reporting among Chinese listed companies and drawing on the approach of Zhang et al. (2023) [40]. First, this study selects the average daily price following the disclosure of financial reports as the market value sampling point. This approach aligns the timing of financial information updates with changes in market value, avoiding excessive corrections caused by market irrationality. It significantly enhances the authenticity of market value feedback on historical financial information and adheres more closely to the principle of the CCA method. Specifically, the stock equity value in the CCA method corresponds to the market value on the first trading day after the financial report is disclosed, provided the daily volatility is less than 2% and matches the latest financial report data. Second, regarding stock price volatility, most existing literature uses arithmetic or moving averages within the reporting period. However, two practical obstacles arise: (1) the time interval between adjacent financial report disclosure dates may be very short (e.g., the annual report of the previous year and the first-quarter report of the current year may differ by only a few days); and (2) even if the time interval between two financial reporting dates is sufficiently long, the effective period of volatility following each report’s release remains a concern. To address these issues, this study proposes a comprehensive method: using the rolling volatility of the previous 30 trading days within 30 trading days after the financial reports are disclosed, followed by rolling volatility over the option cycle. Finally, to mitigate distortions caused by fraudulent financial data, this study evaluates the reliability of financial reports based on the auditor’s opinion. Specifically, financial reports with a "qualified opinion" or "disclaimer of opinion" are flagged as potentially fraudulent, while those with an "unqualified opinion" are deemed reliable. The empirical analysis in subsequent sections demonstrates that conclusions drawn from the financial data without suspected accounting irregularities are more robust than those derived from the entire sample. This finding validates the effectiveness of the auditor’s opinion in correcting the Default Distance.

### 2.2 Validating the effectiveness of the CCA method

**Fig 1:**
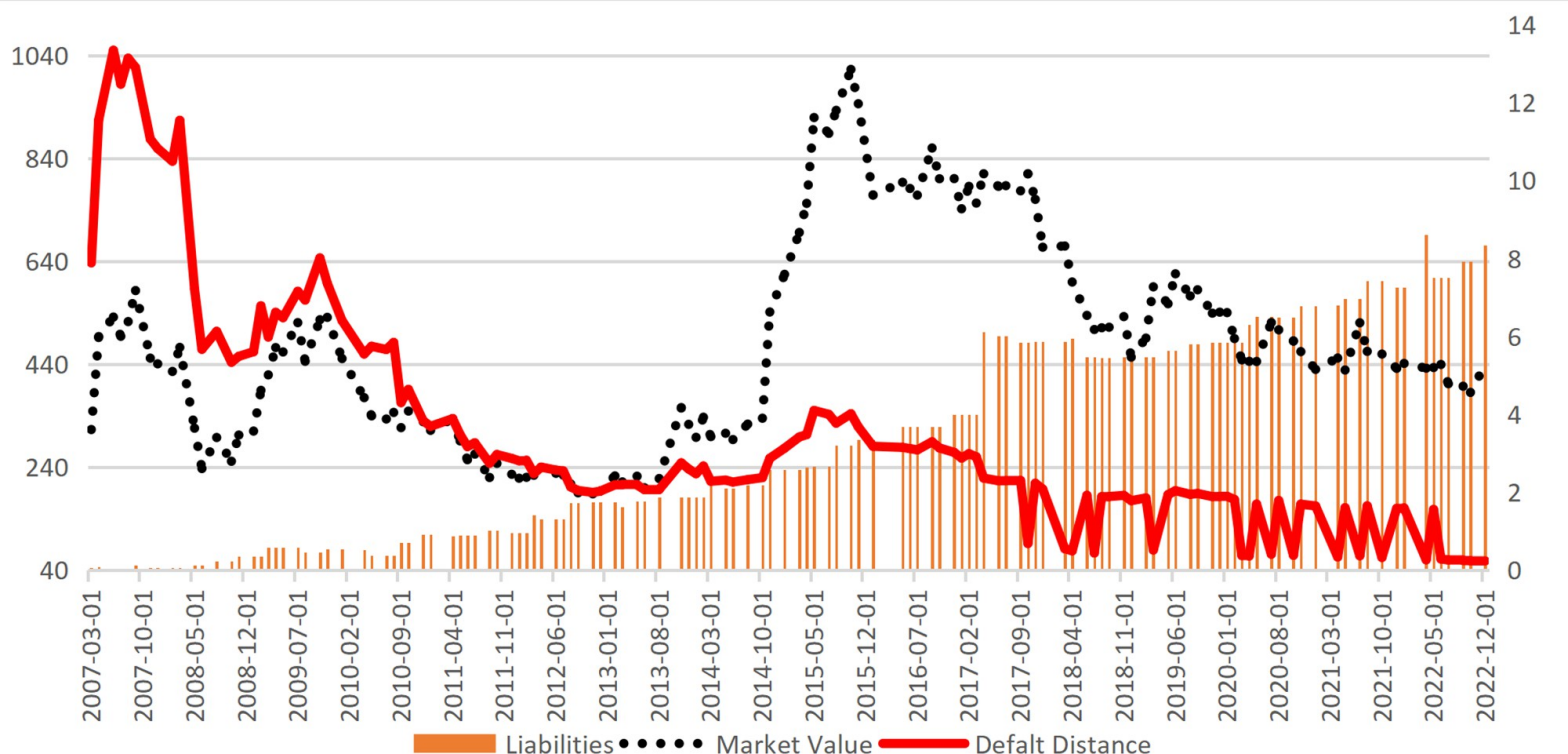
Default distance, market value, and liabilities (Taking Lujiazui as an example).

To preliminarily test the effectiveness of the CCA method, this study takes a renowned construction company, Lujiazui Corporation, as an example. All figures and tables in this study are original creations by the author (The data involved has been provided in Supporting Information, specifically in S1 Data). The analysis examines the relationship between the company’s market value (measured by the closing price), liabilities (derived from the balance sheet items), and the Default Distance over the period from March 2007 to December 2021(Unless otherwise specified, all data utilized in this study are sourced from the Wind database). Fig 1 effectively illustrates the principles of the CCA method: when the company’s liabilities are relatively low(March 2007-June 2012), the changes in the company’s default risk primarily stem from stock price information (the Default Distance closely aligns with market value). However, as the company’s liabilities rise(post-2012), despite experiencing a roller-coaster-like fluctuation in market value, the Default Distance indicates a general downward trend in bankruptcy risk. This suggests that the Default Distance can mitigate the impact of irrational stock price volatility on risk assessments.

**Fig 2:**
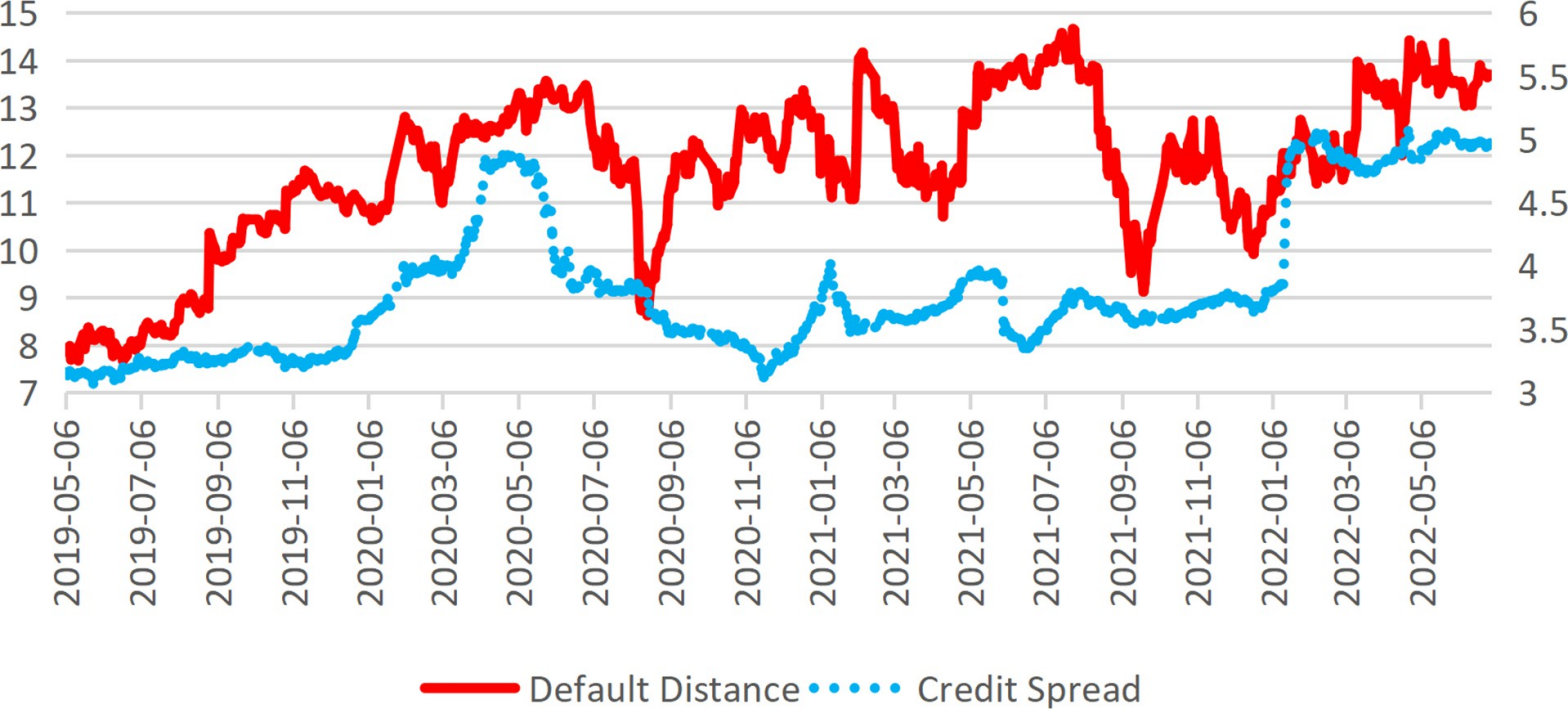
Default distance and credit spread (Taking China nuclear engineering corporation as an example).

This study further validates the effectiveness of the Default Distance as a measure of corporate bankruptcy risk by comparing it with credit spread data. The corporate credit spread is defined as the difference between the yield of its credit bonds and the risk-free rate (represented by the one-year government bond yield). Due to the limited availability of historical credit bond data for publicly listed corporations, particularly those in the construction industry, this analysis focuses on data available from May 2019 onward for comparison with the Default Distance indicator. The results (Fig 2) reveal a strong negative correlation between the two metrics. However, when shifts in risk direction occur, the Default Distance exhibits more pronounced signals with a greater fluctuation amplitude, underscoring its sensitivity in capturing changes in corporate bankruptcy risk.

## 3. Interest rates and bankruptcy risk

**Fig 3:**
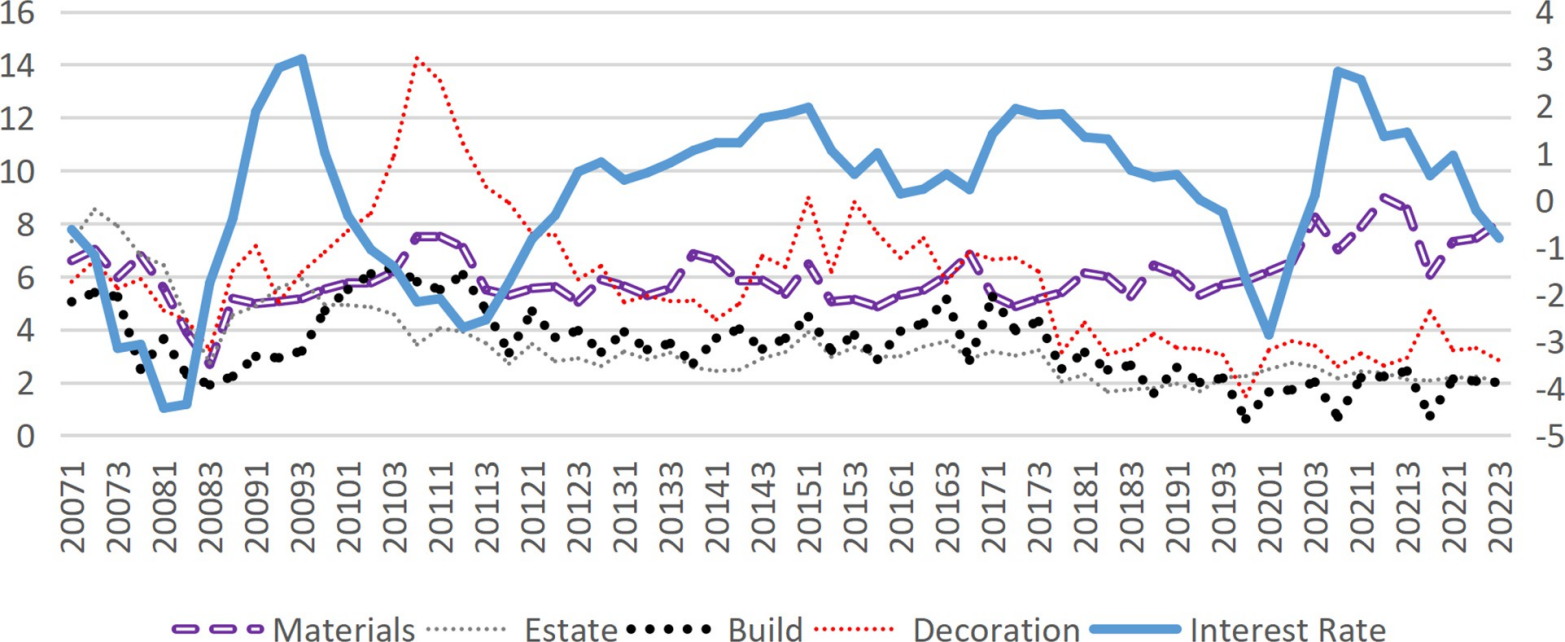
Interest rate and DD of each sub-industry.

This study categorizes the construction industry into upstream and downstream sectors, with the construction industry further segmented into decoration and construction. The upstream industry encompasses the construction materials industry, while the downstream sector includes real estate development and management. Fig 3 demonstrates the significant influence of market interest rate fluctuations(represented by the one-year government bond yield published by the China Monetary Network) on risk within the construction industry. When the industry experiences pronounced effects from interest rate fluctuations, the internal linkage across different sectors intensifies. Although market interest rates primarily represent an exogenous factor affecting construction industry risks, they may also exert influence through certain endogenous mechanisms.

### 3.1 The mediating effect of financing costs

To explore the relationship between market interest rates and bankruptcy risk in construction companies, this study employs an individual fixed effects model. The dependent variable is the company’s Default Distance (DD), while the core explanatory variable is the domestic real market interest rate (InterestRate). Control variables include macroeconomic indicators such as the logarithm of GDP (LnGDP) and the housing sales area (HouseSale), representing the industry’s business climate index. Additionally, the model incorporates firm-specific characteristics that vary over time, such as the logarithm of total assets (LnAsset) and net profit margin (NPM). The descriptive statistics of the main variables, including those involved in the models below, are summarized in Table 1 for convenience. Based on the results of the Hausman test, an individual fixed effects model is chosen for analysis (Note: All models in this study utilize an individual fixed effects model, which will not be mentioned again in subsequent sections).


$${DD}_{it}=\alpha_{0}+\alpha_{1}{InterestRate}_{it}+\alpha_{2}{Controls}_{it}+\mu_{i}+\varepsilon_{it}$$ (9)

Here, *μ*_*i*_ represents the individual fixed effect, and *ε*_*it*_ is the random disturbance term.

**Table 1:** Summary statistics of main variables.

Variable	Variable Declaration	Obs	Mean	SD	Min	Max
DD	Default Distance	13046	3.99	5.45	0.05	48.94
LoanR	Loan financing costs, %	10617	22.76	26.45	0.28	316.73
NPM	Net Profit Margin, %	12901	7.68	19.57	-139.41	125.59
InterestRate	Actual interest rate, %	13316	0.16	1.55	-4.42	3.00
HouseSale	Sales of commercial residential buildings(quarterly growth)	13163	0.06	0.18	-0.31	0.52
LnGDP	Log(GDP), ¥100 million	13163	11.91	0.47	10.89	12.63
LnAsset	Log(total assets), ¥100 million	13163	13.71	1.67	5.02	19.39
ROA	Return on total assets, %	12901	2.86	3.13	-5.16	16.68
Loan	Loan financing accounts for external financing	12028	0.67	0.28	0.00	1.00
SCF	Supply chain financing accounts for external financing	11788	0.09	0.87	-8.79	0.99
Capital	Capital market financing accounts for external financing	12028	0.02	0.08	0.00	1.00
KZs	Financing constraint index	6722	-1.54	0.97	-5.37	0.15
HHI	Herfindahl index	13163	0.09	0.05	0.03	0.21
PM	Building materials price index	8291	133.42	28.79	79.80	203.32
avDD	The industry average of DD	13316	3.99	1.86	0.62	14.24
avLoanR	The industry average of LoanR	13163	0.15	0.15	0.00	1.00

The regression results (Table 2) reveal a negative relationship between interest rates and default risk: as interest rates rise, the Default Distance decreases, indicating a heightened bankruptcy risk for companies. The transition from Model 1 to Model 2 highlights the endogeneity issue caused by omitted variables. This finding highlights the risk contagion effect of interest rate fluctuations on the construction industry’s demand chain. As a critical macroeconomic variable, interest rates substantially influence the national economy and household income, thereby impacting the industry’s demand-side risk. After accounting for macroeconomic variables (LnGDP) and industry demand (HouseSale), the magnitude of its effect diminishes, with the coefficient decreasing from -0.314 in Model 1 to -0.0901 in Model 2. Additionally, these two demand-related variables may positively influence the construction industry through their exogenous variations, as indicated by the coefficient signs aligning with economic intuition.

**Table 2:** Regression results of default risk on interest rate.

	(1)	(2)	(3)	(4)	(5)	(6)
VARIABLES	DD	DD	DD	LoanR	DD	NPM
InterestRate	-0.314***	-0.0901***	-0.0477***	0.433***		
LoanR			-0.00261**		-0.00259**	-0.0432***
HouseSale		1.584***	1.245***	-8.513***	1.189***	5.169***
LnGDP		1.712***	1.315***		1.260***	-2.243***
LnAsset		-2.577***	-2.221***	-0.316	-2.224***	0.946***
NPM	0.0278***	0.0308***	0.0213***	-0.108***	0.0213***	
Constant	3.766***	18.62***	18.08***	27.57***	18.78***	20.97***
Observations	12,666	12,666	9,147	9,217	9,147	9,217
R-squared	0.031	0.236	0.267	0.010	0.266	0.011
Sample	283	283	269	269	269	269

**Note(s):** Standard errors in parentheses.

*** p<0.01

** p<0.05

* p<0.1

**Fig 4:**
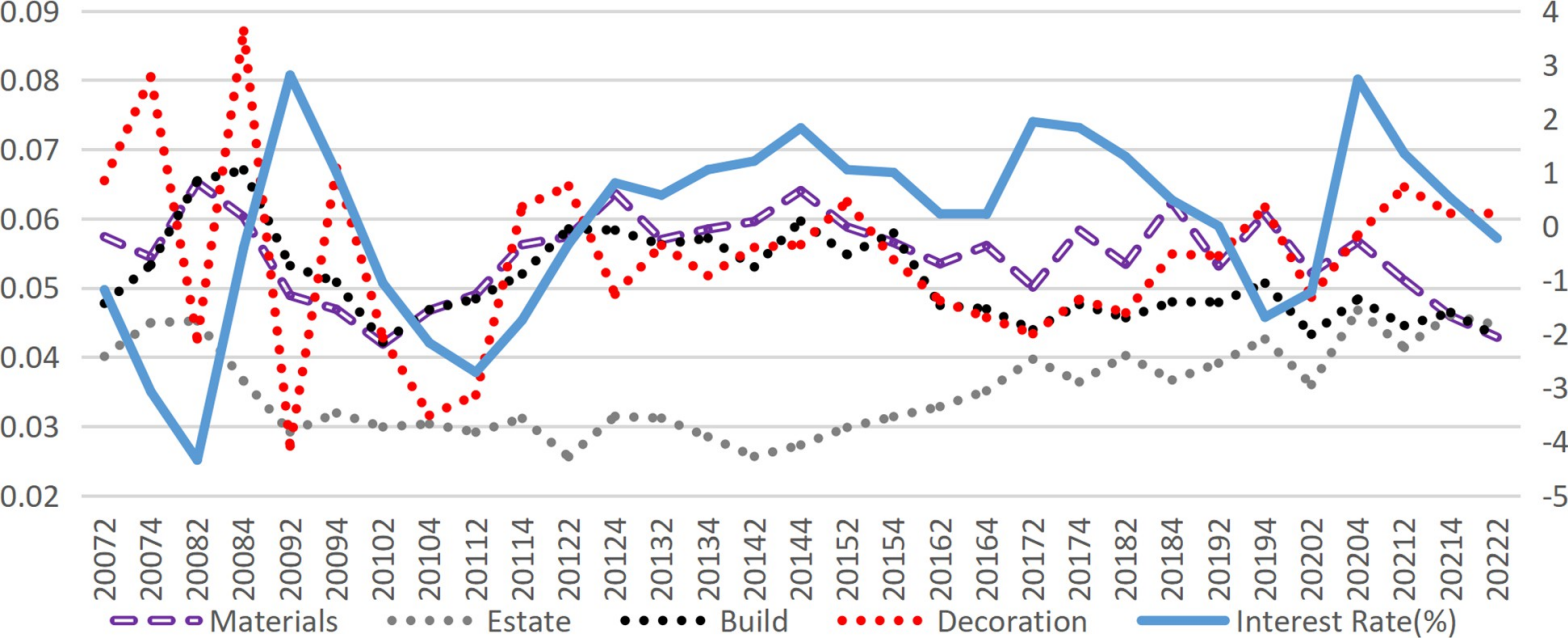
Market interest rate and the interest-bearing liability interest rate of each sub-industry in the construction industry.

According to the credit transmission theory of monetary policy, an increase in market interest rates raises the financing costs for enterprises. These financing costs directly affect the operating profit of enterprises, which subsequently impacts their bankruptcy risk. Therefore, the most immediate channel through which interest rates impact corporate default risk is via credit costs. In this study, the interest expense of construction companies is divided by their interest-bearing debt to approximate the interest-bearing debt rate, which serves as a proxy for the company’s credit financing rate (Given the minimal share of bond financing within interest-bearing debt, this estimate effectively reflects trends in credit financing rates.). After incorporating the credit financing rate into Model 2, the effect of interest rates further diminished, with the regression coefficient decreasing from -0.0901 to -0.0477. However, it remained significant and had a positive effect on the credit financing rate (Model 4), suggesting the presence of mediation effects. Calculations reveal that the mediation effect of financing costs is only 1.26% (derived from Model 3 and Model 4), indicating that the influence of raising interest rates on default risk via increased financing costs is relatively limited. The actual interest rates are typically observed within the range of -0.813% to 1.29% (excluding extreme values in the top and bottom 25%). Nevertheless, raising interest rates demonstrably reduces corporate profitability, as supported by real-world evidence and prior research [41]. As illustrated in Fig 4, the median interest rates and credit financing rates for various sub-sectors of construction remain highly correlated. Furthermore, Model 6 confirms that higher market interest rates significantly lower the net profit margin of enterprises. Tests indicate that the mediation effect of the credit financing rates in the impact of interest rates on the net profit margin of enterprises is substantial, accounting for 33.63%.

Building on the preceding analysis, this study proposes a hypothesis: enterprises modify their operations to prioritize profitability when faced with rising rates, thereby increasing their risk exposure. In essence, the rise in interest rates compels enterprises to sacrifice safety for profitability. Beyond the influence of credit costs, as discussed in the literature review, market interest rate fluctuations also impact enterprises through changes in financing structure and capital redistribution. The subsequent section explores how these two additional factors contribute to bankruptcy risk under varying interest rate conditions.

### 3.2 Short-term effect: Transformation of financing structure

Enterprise financing can be broadly categorized into internal and external financing sources. Internal financing primarily depends on retained earnings as the source of capital for reproduction. However, regardless of an enterprise’s size or operating mechanism, external financing is more sensitive to market interest rate fluctuations. External financing encompasses several channels, including credit financing, supply chain financing (commercial credit), and capital market financing (such as equity and bond issuance). Market interest rate fluctuations significantly influence the financing structure of enterprises. When interest rates rise, the cost of credit financing increases substantially, pressuring enterprises to reevaluate and adjust their financing structures. The primary objective is to balance two critical needs: securing sufficient capital for operations and reproduction (capital quantity) while stabilizing total financing costs (capital price). This dual challenge compels firms to explore alternative financing options or optimize their reliance on existing channels to mitigate the financial strain induced by higher interest rates.

**Fig 5:**
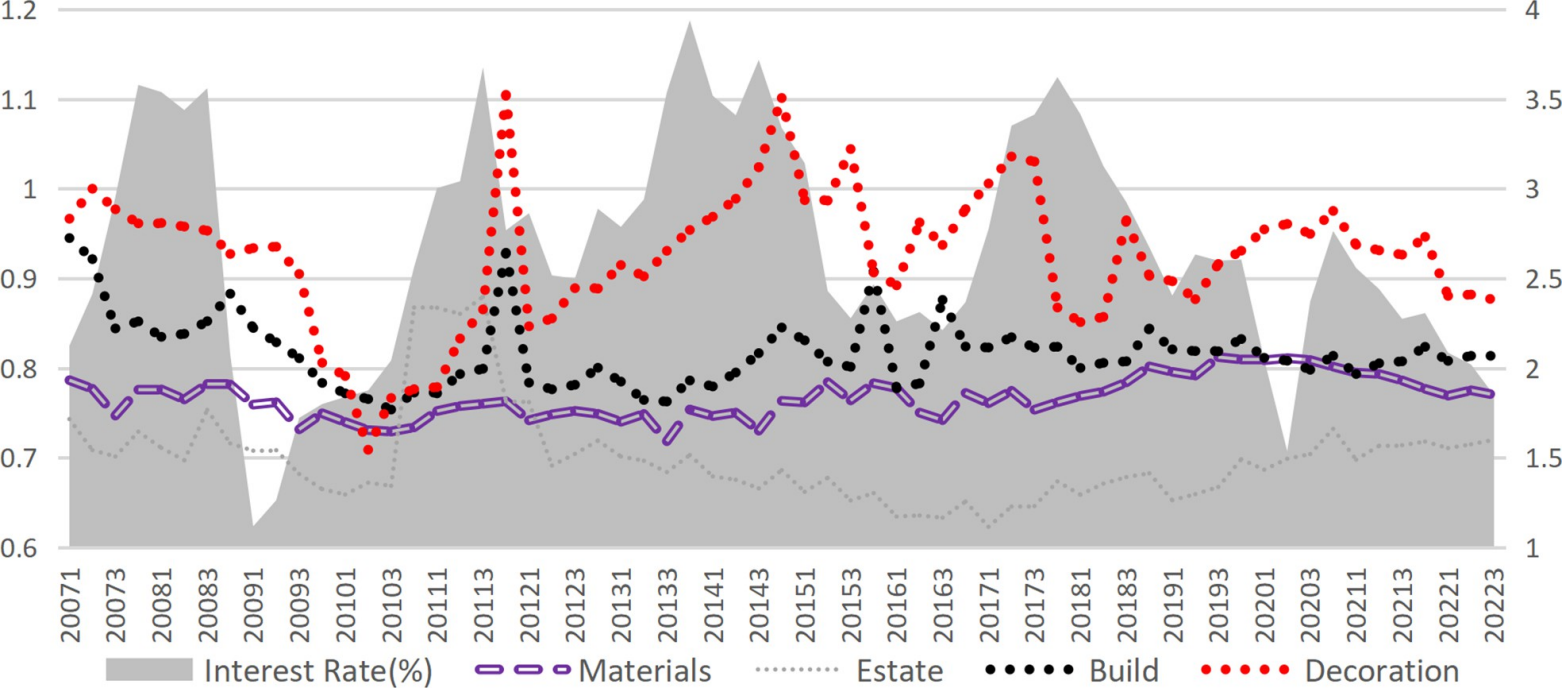
Proportion of current liabilities in total liabilities.

Due to the underdeveloped capital market in China, supply chain financing is the most prominent external financing method after credit financing, offering a lower-cost alternative. Existing literature has extensively analyzed how increased reliance on supply chain financing can enhance enterprise performance [42]. Consequently, when market interest rates rise, enterprises tend to increase their use of supply chain financing to offset the rising costs of credit financing. However, supply chain financing is typically classified as short-term debt, which results in a higher proportion of short-term liabilities. Given that enterprise investment activities are generally inflexible in the short term, this shift weakens firms’ debt-servicing capacity. It is worth noting that maturity mismatch between investment and financing is a critical factor contributing to liquidity shortages and eventual debt defaults [43, 44]. As illustrated in Fig 5, a significant positive correlation exists between the proportion of short-term liabilities and market interest rates. On the other hand, supply chain financing, as commercial credit within the industry, can improve a firm’s financial conditions when utilized judiciously [45]. However, excessive dependence on supply chain financing amplifies risk interconnectivity among firms in the same industry, thereby increasing the potential for cascading failures across the entire industry chain [46].

#### 3.2.1 The structural characteristics of financing in the construction industry

Data specification. An enterprise’s supply chain financing is the sum of commercial credit financing from its upstream and downstream enterprises. The financing amount from upstream enterprises is derived by subtracting *advances received* from accounts payable and *notes payable* in the financial statements of the company. In contrast, the financing amount from downstream enterprises is determined by subtracting *accounts receivable* and *promissory notes* from *advances received*. Credit financing refers to the total amount of borrowing projects (including short-term and long-term borrowings), mainly from bank loans. Capital market financing encompasses the issuance of bonds and stocks publicly during the reporting period. All data used in this study are obtained from the Wind database and financial reports published by listed companies.

**Fig 6:**
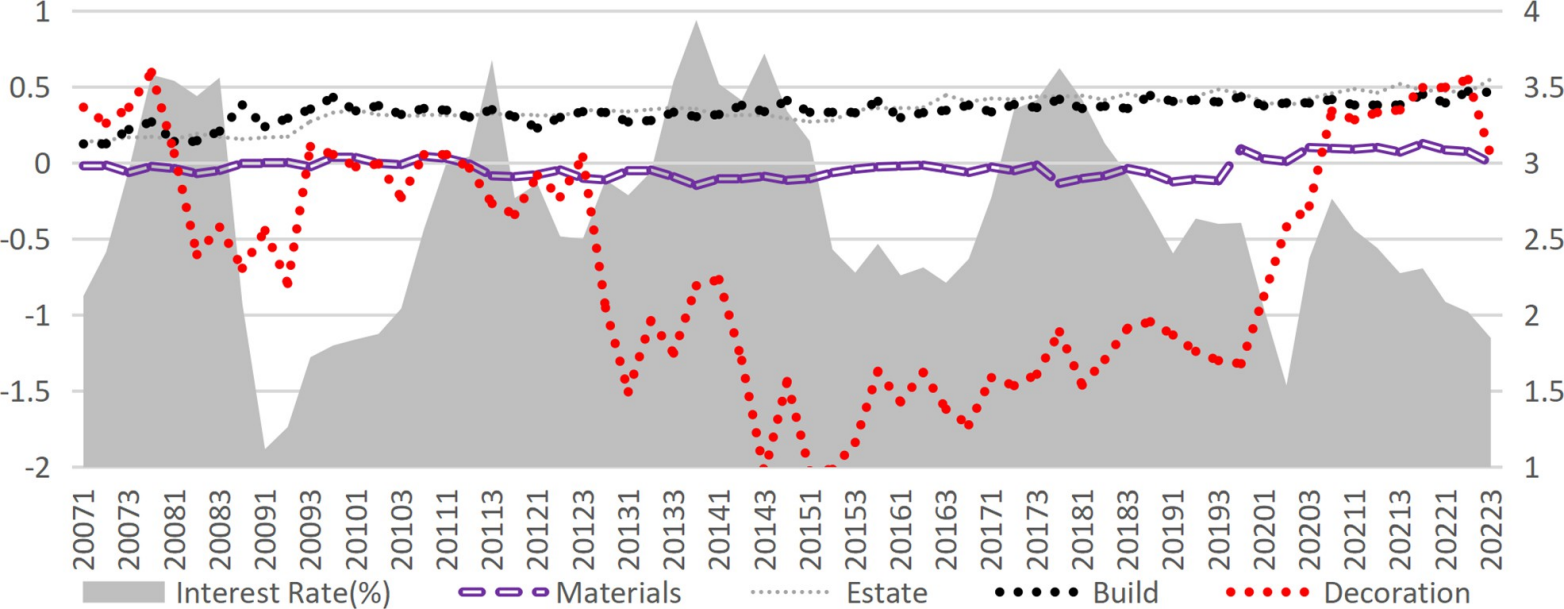
Supply chain financing accounts for external financing.

**Fig 7:**
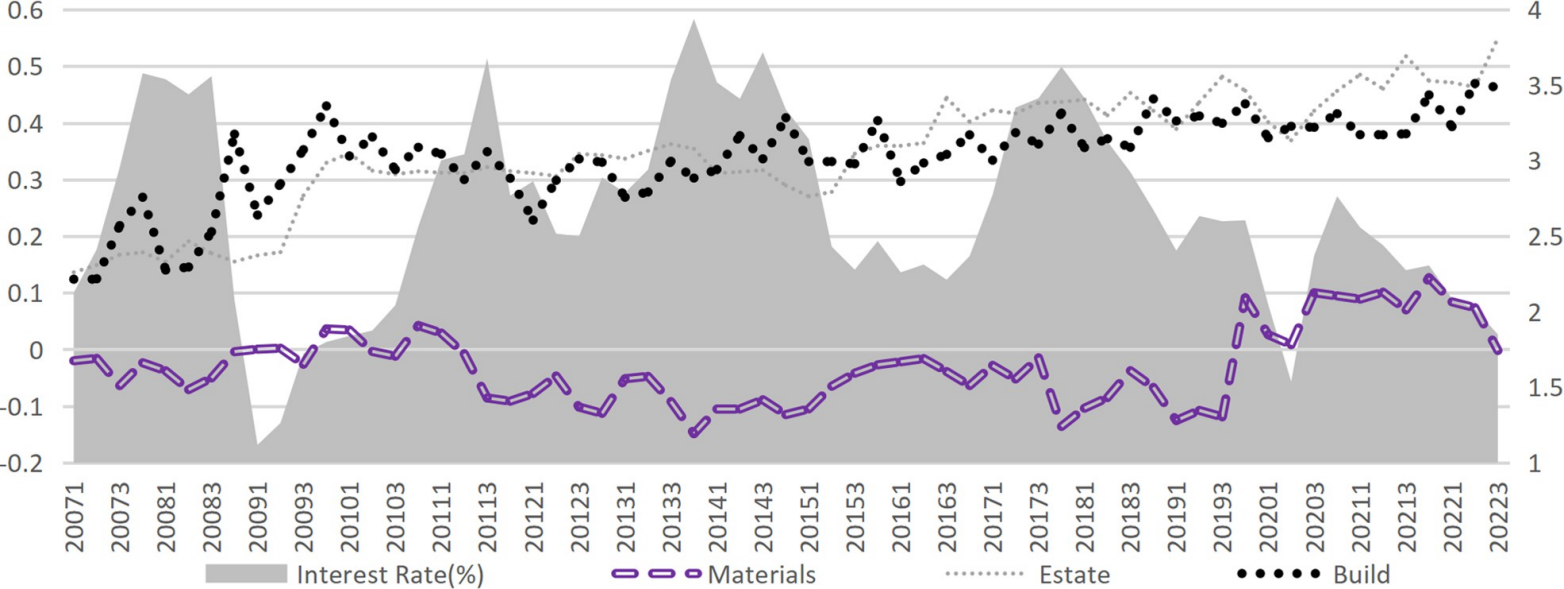
Supply chain financing accounts for external financing (excluding the decoration industry).

**Fig 8:**
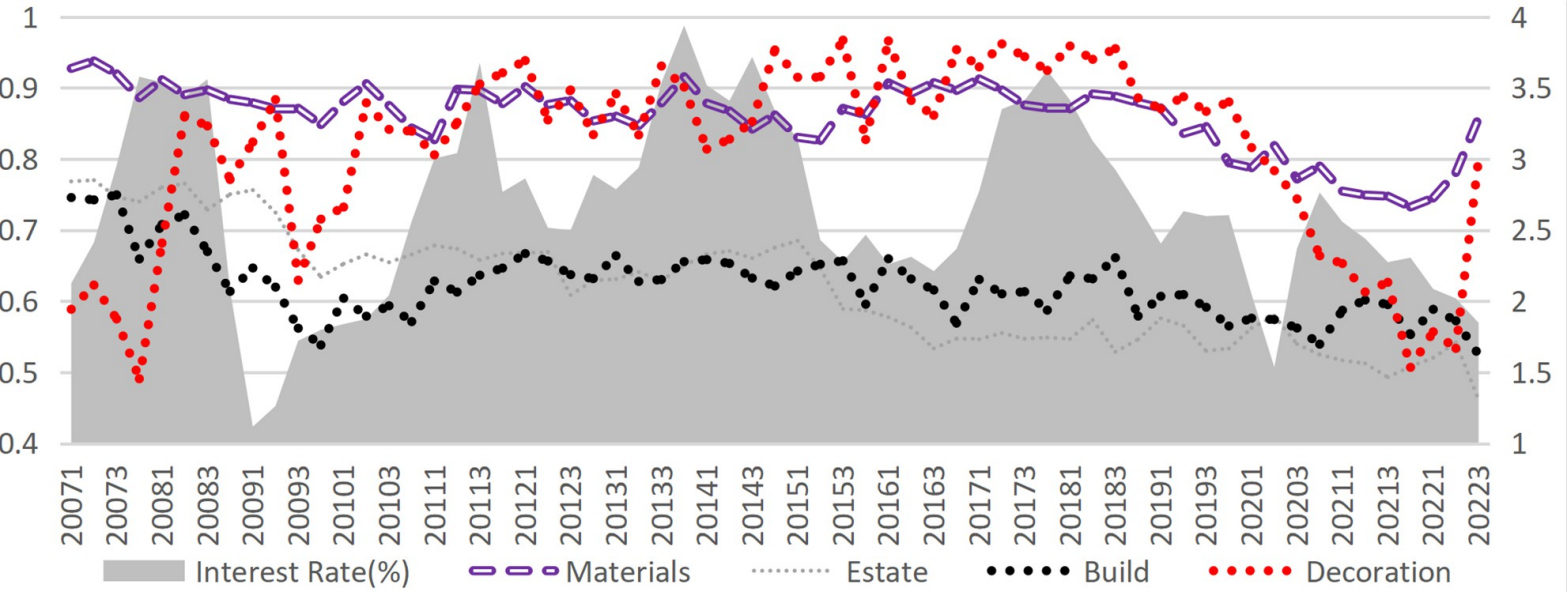
Credit financing accounts for external financing.

**Fig 9:**
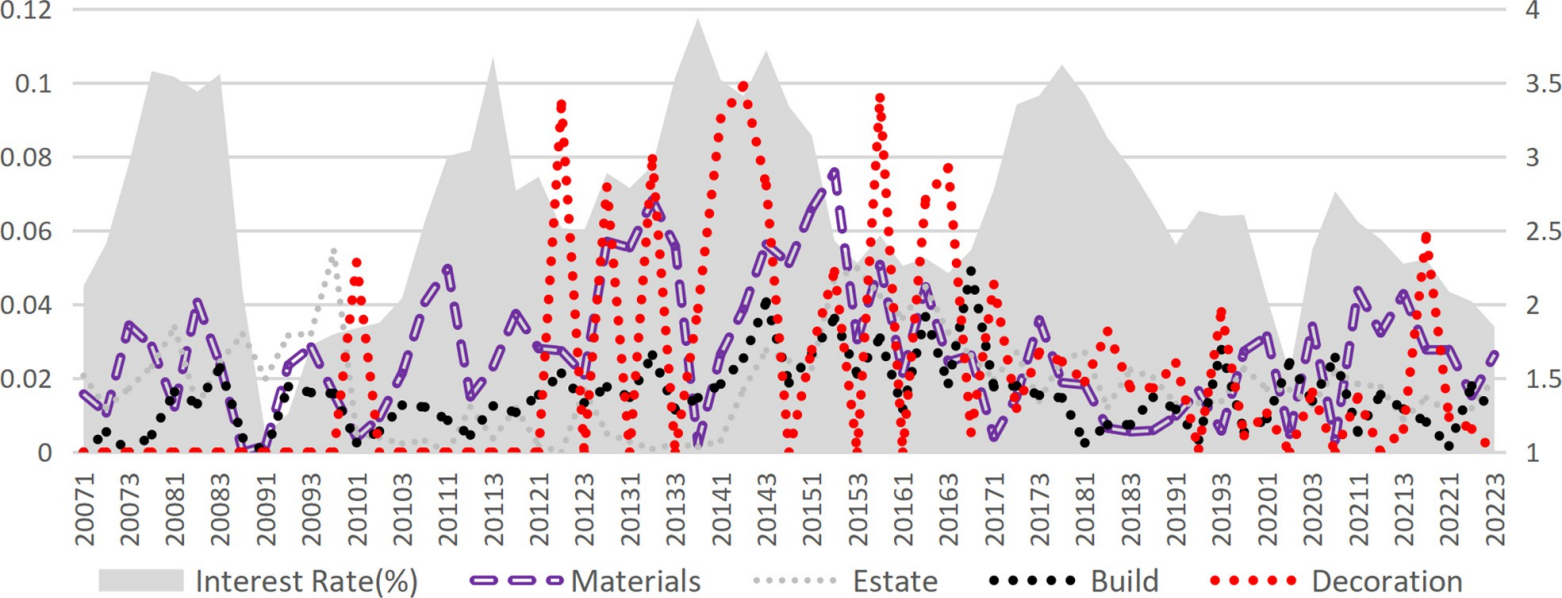
Capital market financing accounts for external financing.

**Fig 10:**
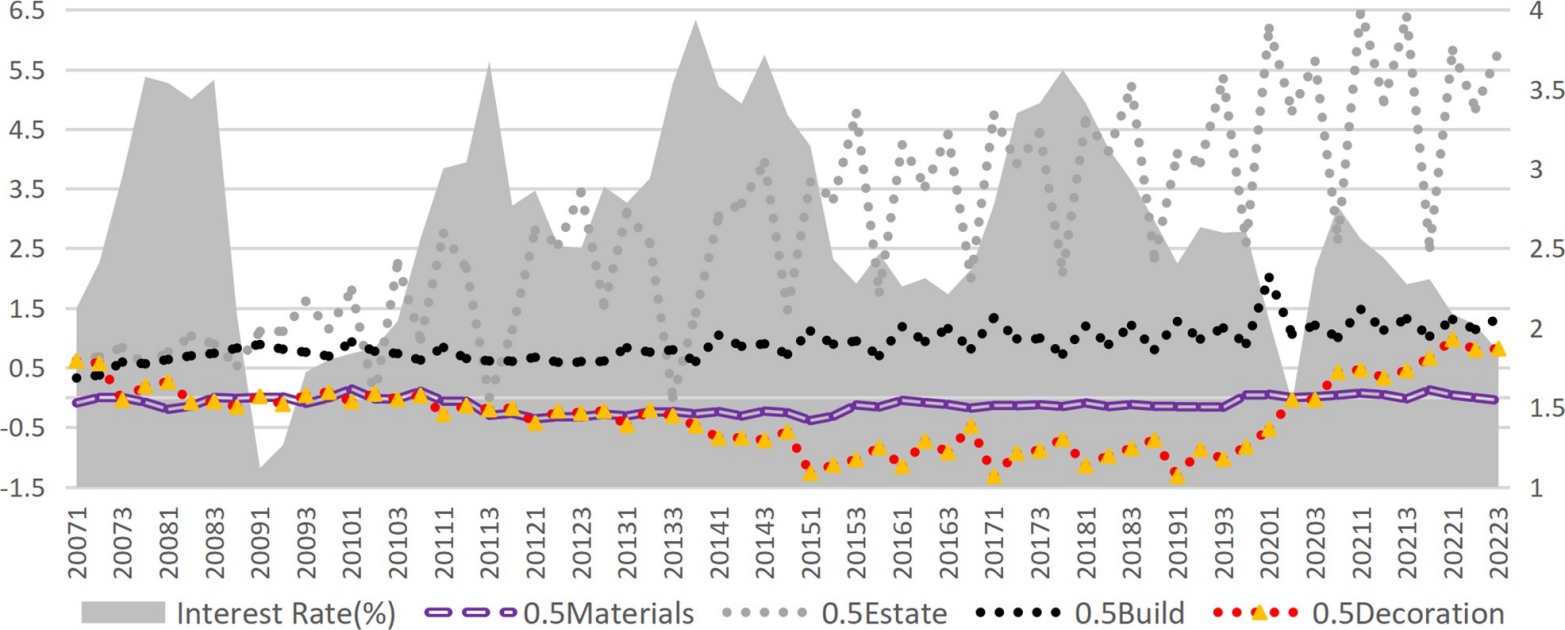
Supply chain financing accounts for revenue.

**Fig 11:**
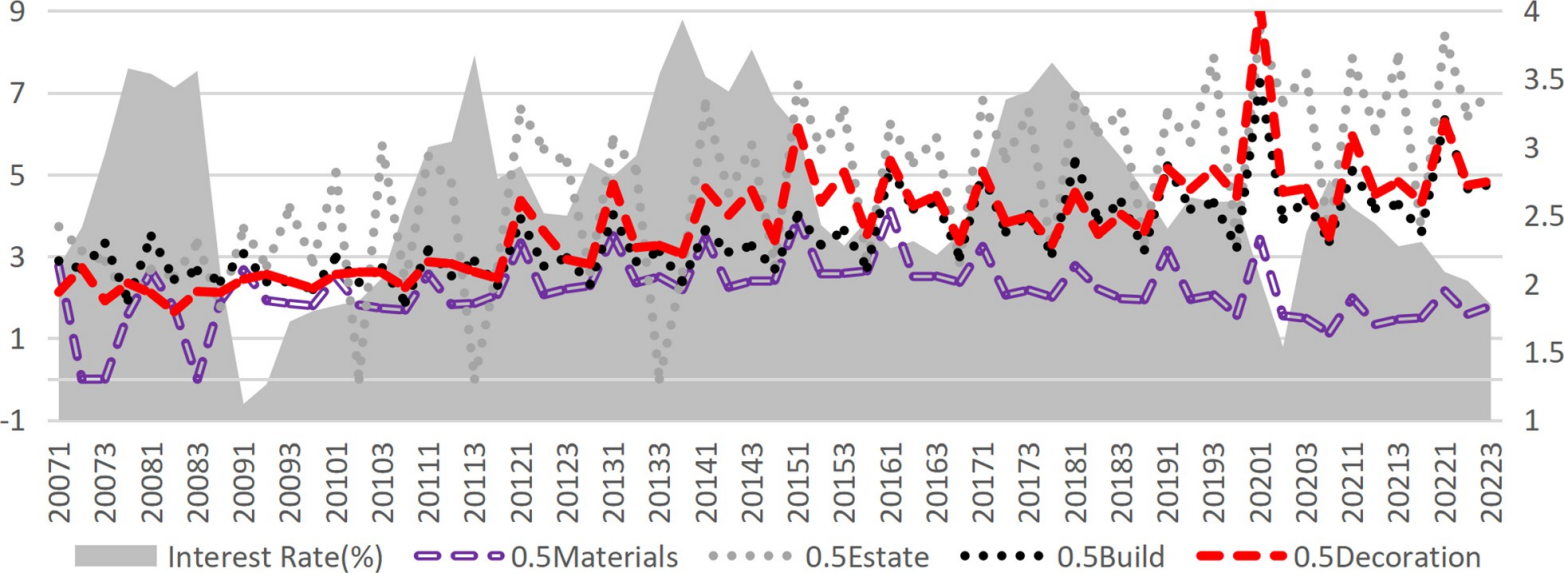
Total supply chain financing of industry (the sum of absolute values of supply chain financing accounts for revenue).

External financing structure. An analysis of the three external financing channels in the construction industry (Figs 6–9) reveals that credit financing serves as the dominant channel, while capital market financing remains minimal (with most proportions below 5% and a maximum not exceeding 11%). Significant differences exist in the utilization of supply chain financing across various industries. Notably, since 2020, the proportion of supply chain financing has surged across sectors, particularly in the decoration and building materials industry. Supply chain financing in the building materials industry has turned positive since 2020, while the decoration industry has experienced a remarkable shift: transitioning from negative values in Q3 2020 to positive values by 2021, where it even surpassed credit financing to become the most critical external financing channel. Compared to the upstream industries in the construction industry (building materials and decoration industry), downstream industries (construction and real estate industries) can obtain more funds from the supply chain (Fig 10). The financing obtained by the real estate industry from upstream and downstream is several times greater than its operating income, occupying an apparent advantageous position in the industrial chain trade. The supply chain financing ability of the construction industry ranks second but is far behind the real estate industry. In contrast, the decoration and building materials industries have the lowest level of supply chain financing (Fig 11).

Financing structure and interest rates. Supply chain financing and capital market financing, as alternative tools to credit financing, generally exhibit a positive correlation with interest rate fluctuations. However, this relationship is not consistently significant across all periods, indicating that additional factors to be controlled may influence the interplay between interest rate changes and adjustments in financing structure. On the other hand, the sensitivity to interest rate fluctuations also varies among sub-industries. The decoration industry demonstrates the highest sensitivity, likely due to its relatively low-profit margin. According to statistics, the median net profit margin of decoration enterprises in the sample set is only 4.03%, compared to 8.15% for other industries. This evidence suggests that the rise in interest rates increases the cost of credit financing, eroding profitability and prompting enterprises to substitute some credit financing with supply chain financing. Furthermore, such "stimulus-response" behavior is particularly pronounced among enterprises with weaker, underscoring the role of financial constraints in shaping financing decisions under changing interest rate conditions.

#### 3.2.2 Modeling analysis of short-term effects

In the short term, enterprises cannot adjust their scale, and the optimal condition for profit maximization is where marginal cost equals marginal revenue. When interest rates rise, both marginal and average costs increase. Due to the inability to alter their scale in the short term, firms in monopolistically competitive industries may face losses(at least reduced profits) in the short term. To cope with this dire situation, enterprises adjust their financing structure to hedge or mitigate the risk of rising interest rates to increase costs. This study introduces the financing structure as a short-term adjustable variable under the theoretical framework of market structure and firm behavior. This addition enables a deeper exploration of the interplay between market interest rate, financing structure, and bankruptcy risk. In short-term equilibrium, the profit maximization condition that marginal revenue equals marginal cost determines an enterprise’s optimal output and optimal cost. Consequently, the corresponding financing costs attains a specific optimal value. When market interest rates fluctuate, they primarily affect credit costs. Enterprises can quickly offset the impacts by altering their financing structure. Financing costs are predominantly composed of supply chain and credit financing (capital market financing requires a lengthy application and queuing process and is difficult to obtain in the short term). Accordingly, the total financing costs can be expressed by the following formula:

$$r_{All}=\alpha_{SCF}*r_{SCF}+(1-\alpha_{SCF})*r_{Loan}$$ (10)

Among them, *r*_*All*_ refers to the total financing interest rate, which is the specific optimal value. *α*_*SCF*_ is the proportion of supply chain financing in the total external financing. *r*_*SCF*_ and *r*_*Loan*_ are the supply chain financing costs rate and credit financing interest rates, respectively.


$$\alpha_{SCF}=\frac{r_{Loan}-r_{All}}{r_{Loan}-r_{SCF}}=1-\frac{r_{All}-r_{SCF}}{r_{Loan}-r_{SCF}}$$ (11)

In the short term, assuming that *r*_*All*_ and *r*_*SCF*_ remain unchanged, a rise in market interest rates directly increases financing costs. Thus, *α*_*SCF*_ increases, and ultimately, the proportion of current liabilities in the total liabilities increases (because the term of supply chain financing is shorter than credit financing). Within the framework of CCA, the composition of liabilities(*K*_*t*_) can be expressed as:

$$K_{t}={CL}_{t}+\beta *{NCL}_{t}$$ (12))

Here, *CL*_*t*_ and *NCL*_*t*_ are current and non-current liabilities, respectively, and *β* is the debt conversion factor of non-current liabilities, generally between 0 and 1. Therefore, keeping the sum of *CL*_*t*_ and *NCL*_*t*_ unchanged, *K*_*t*_ will become larger when the proportion of *CL*_*t*_ increases. Combined with the enterprise default probability (Probability of Default, PD), the consequence of increasing *K*_*t*_ is that the default probability increases, that is, the risk of bankruptcy increases.


$${PD}_{t}(r_{Loan})={PD}_{t}(\alpha_{SCF})={PD}_{t}(K_{t})=N\left(-\frac{ln(S_{0}/K_{t})+(r_{t}-{\sigma_{t}}^{2}/2)(T-t)}{\sigma_{t}\sigma \sqrt{T-t}}\right)$$ (13)

Empirical testing is grounded in the theoretical framework and empirical data discussed above. To evaluate the influence of the financing structure on the risk of enterprise bankruptcy, it is crucial to control the enterprise’s financing ability, thereby mitigating endogeneity concerns. This study incorporates a vital control variable: the financing constraint index (KZs). Originally developed by Kaplan and Zingales [47] (1995) [47], KZs has been adapted by numerous Chinese scholars [48, 49] to align with Chinese enterprise data. This study adopts the coefficients of Li Junping and Xu Longbing (2015) [49] to construct KZs. As shown in the regression results (Table 3), rising interest rates lead to a decrease in the proportion of credit financing while increasing the proportion of supply chain financing and capital market(Model 1-Model 3). This demonstrates the significant impact of interest rate adjustments on the financing structure. However, the rising effect of supply chain financing surpasses that of capital market financing, as reflected in the coefficients. This disparity arises from the relative proportion of these two external financing channels (Figs 6 and 9). The limited increase in capital market financing stems from both subjective and objective constraints. For capital market financing, enterprises not only need to have some objective and rigid conditions (such as profit for several consecutive years, in line with the government’s industrial policies), but also have subjective obstacles (such as the reluctance of original shareholders to relinquish control in equity financing). Model 4-Model 6 explore the impact of the three financing methods on enterprise default risk. Under otherwise unchanged conditions, a higher proportion of supply chain financing and credit financing increases risk, while a greater reliance on capital market financing mitigates it. Model 7 suggests that a higher proportion of supply chain financing harms the profitability of enterprises. This result appears contradictory to the earlier analysis, where increasing supply chain financing during rising interest rates mitigates short-term profit declines. By integrating interest rate changes in Model 8, it becomes evident that: (1) Supply chain financing exerts a more substantial direct negative impact on profitability (coefficient changes from -0.202 to -0.237); (2) A positive effect of 0.035 emerges indirectly through the transmission effect of interest rate changes. Supply chain financing accounts for a more considerable negative impact on corporate profitability without considering the intermediary effect of interest rate fluctuations (control the influence of interest rate fluctuations in Model 8). Nevertheless, considering the change in the financing structure (total financing costs fell), the supply chain financing negative effect on profits (Model 7) fell. In addition, the negative impact of the proportion of supply chain financing on corporate profitability can be explained as the limited volume of supply chain financing (goods transactions limit commercial credit). When enterprises rely too much on supply chain financing, their operating efficiency will decline(To get the other side to provide supply chain financing, some preferential terms must be transferred). Only when rising interest rates hinder corporate credit financing, choosing more supply chain financing will partially mitigate the negative impact of rising interest rates.

**Table 3:** Interest rate, enterprise bankruptcy risk, and financing structure.

	(1)	(2)	(3)	(4)	(5)	(6)	(7)	(8)
Variables	Loan	SCF	Capital	DD	DD	DD	ROA	ROA
InterestRate	-0.0107 ***	0.0139***	0.00154***					-0.237***
SCF				-0.610***			-0.202 ***	-0.219***
Loan					-0.433**			
Capital						1.568 ***		
HouseSale				1.347 ***	1.326 ***	1.298 ***	0.954 ***	1.305 ***
LnAsset				-1.894***	-2.028***	-2.040***		
NPM				0.0274***	0.0286***	0.0287***		
LnGDP				1.266 ***	1.196 ***	1.268 ***	-0.931 ***	-0.676***
KZs				-0.114**	-0.142***	-0.145***	-1.392 ***	-1.405***
Constant	0.672 ***	0.0925***	0.0200 ***	13.85 ***	16.75 ***	15.74 ***	12.65 ***	9.627 ***
Sample	281	280	281	267	270	270	267	267

**Note(s):S**tandard errors in parentheses

*** p<0.01

** p<0.05

* p<0.1

### 3.3 Long-term effect: Transformation of industry competitiveness

In the short term, enterprises can only adjust their financing structure in response to interest rate changes to stabilize financing costs and maintain financing volumes. However, in the long term, interest rate fluctuations eventually influence reproduction levels through changes in total financing costs. Enterprises make investment decisions based on rational expectations of interest rates, with enterprises at the industry-leading level being more inclined to expand their scale under low interest rates to consolidate or increase market share, thereby driving higher industry concentration. Studies have demonstrated that [50] declining interest rates exert a more significant impact on monopolistic and competitive industries, as leaders are incentivized to borrow and invest to sustain or strengthen their market dominance [50].

**Fig 12:**
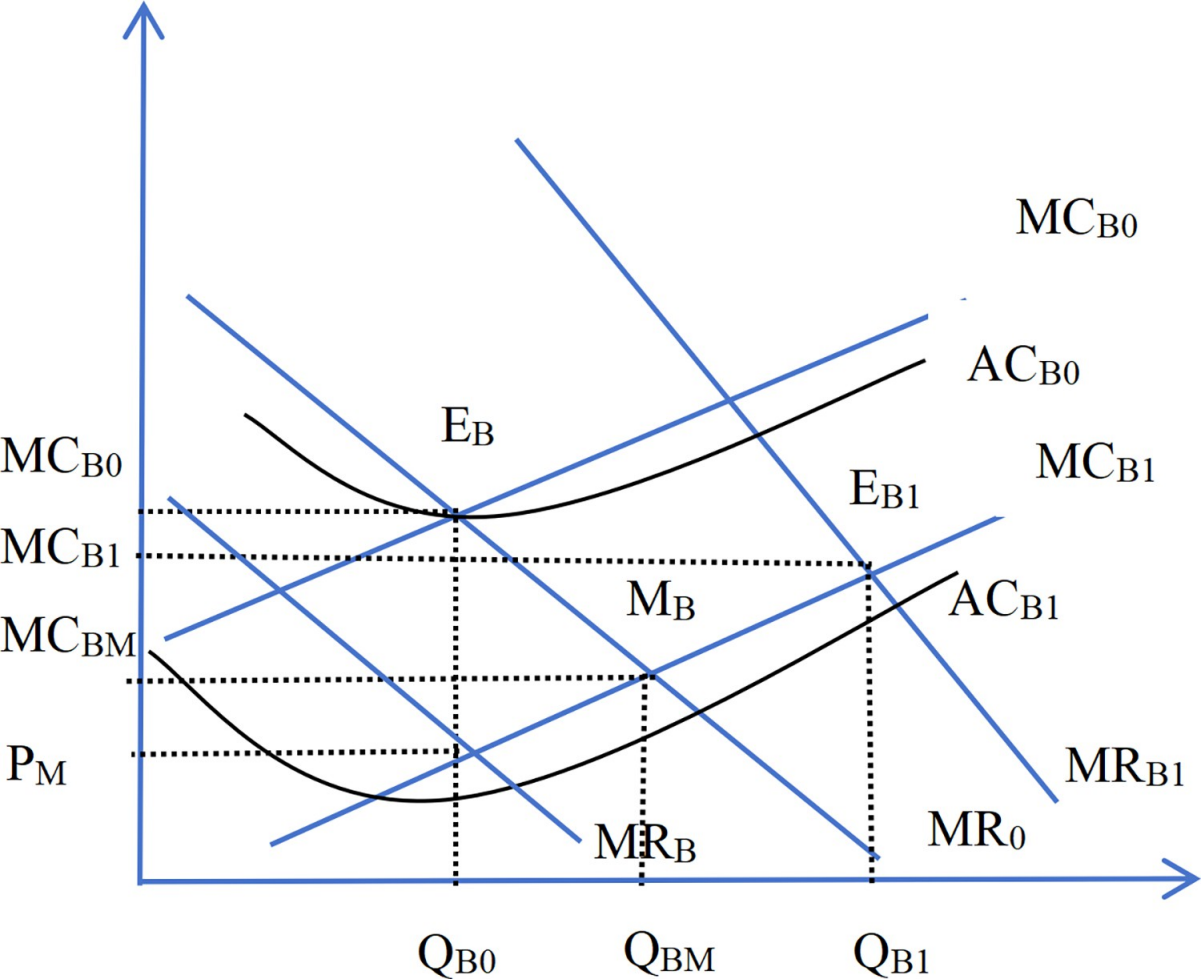
The production of big enterprises after the decline of the interest rate.

**Fig 13:**
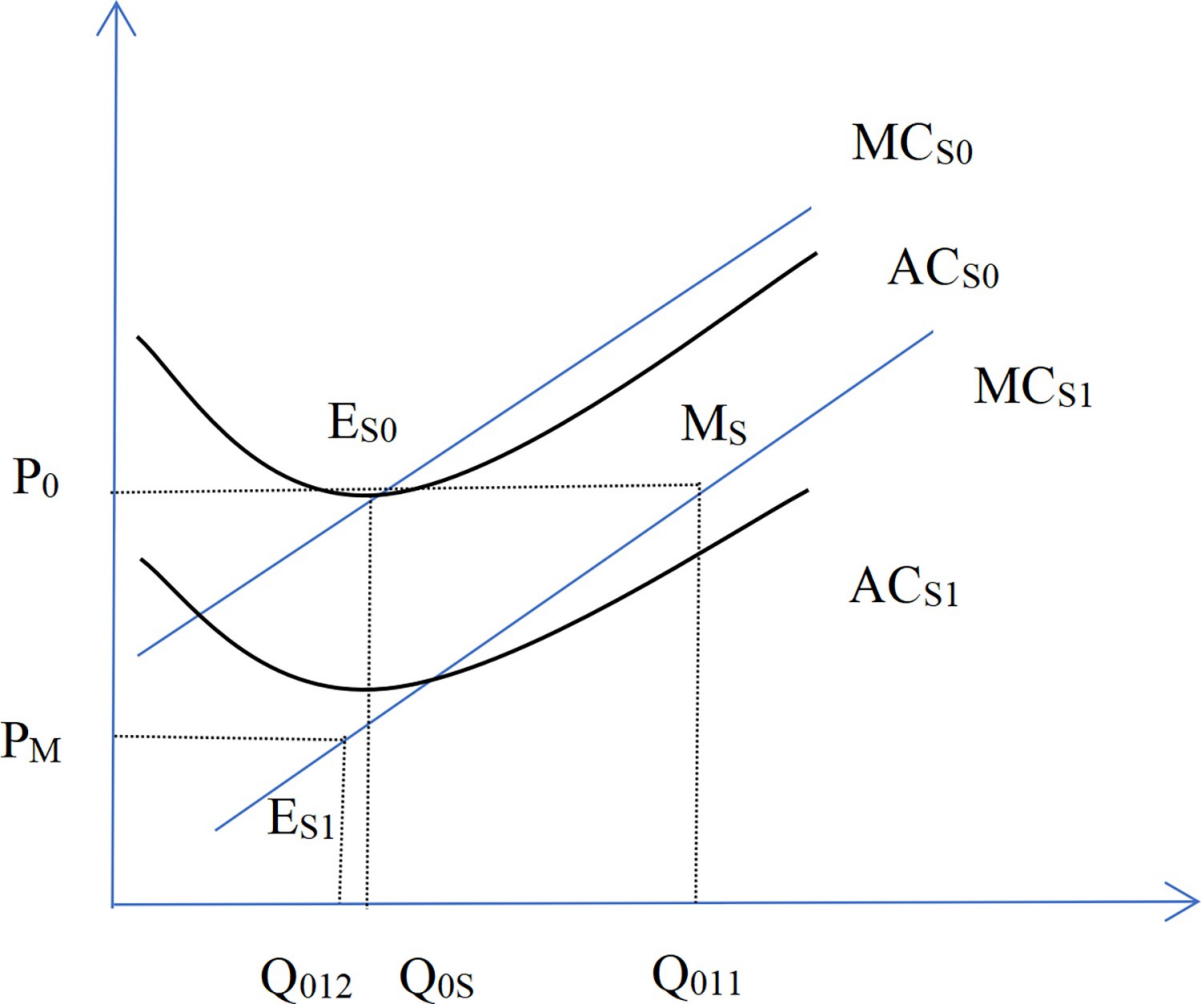
Production of small enterprises after the decline of interest rates.

**Fig 14:**
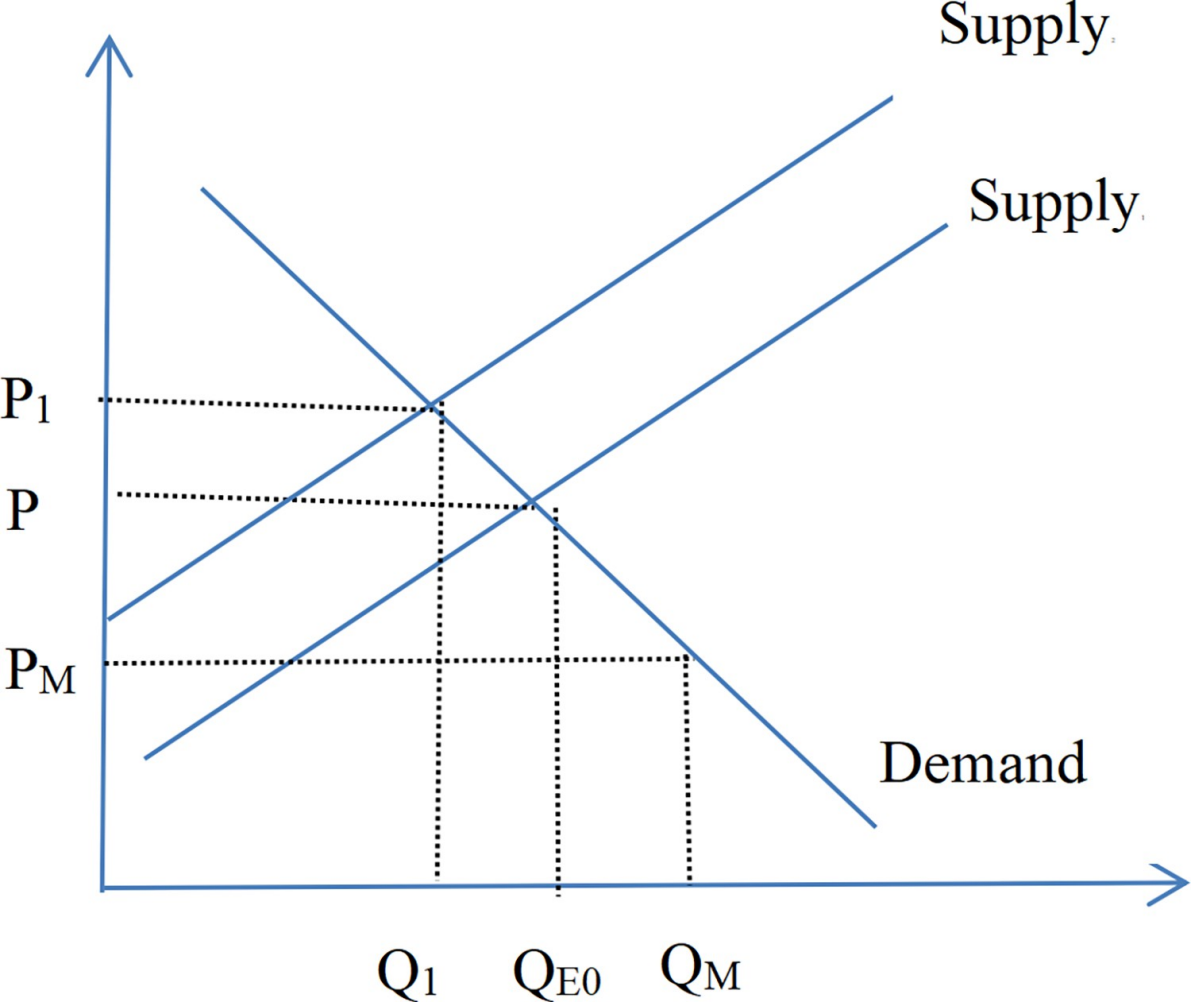
The market supply and demand after the decline of interest rates.

The following section presents a theoretical derivation of the mechanism by which market interest rate influence the degree of industry agglomeration (or monopoly). Figs 12 and 13 illustrate that large enterprises(denoted by the subscript B) possess some market power, resulting in their marginal return curve sloping downward to the right bottom. In contrast, small enterprises (denoted by the subscript S) exert minimal influence on the market price, as their role as price takers aligns their marginal return curve closely with the horizontal line representing the market price. A reduction in market interest rate decreases both the average cost and the marginal cost of financing costs. This study delineates this process into three stages. In the first phase, production has soared. When the interest rates decline, the equilibrium point from E_B0_ (E_S0_ for small enterprises) to M_B_(M_S_ for small enterprises) reflects increased production driven by reduced marginal cost. The second phase is price plummets. An increase in production leads to oversupply, causing market prices to drop from P_0_ to P_M_ (Fig 14). Due to their cost advantage, leading companies are less affected by falling prices, while small enterprises—facing steeper marginal cost curves—experience greater profit erosion. As prices dip below average costs, smaller firms may exit the market. Conversely, large enterprises, with their flatter marginal cost curves, maintain profitability as their marginal return curve shifts inward from MR_0_ inside to MR_BM_. In the third stage, the industry has gathered. Under the pressure of falling prices, small enterprises experience declining profits or incur losses. Large enterprises leverage their cost and operating advantages to capture the vacated market share, increasing industry concentration. As large enterprises gain greater market dominance, their marginal return curve shifts outward to MR_B1_, leading to a new equilibrium at E_B1_ with higher profitability. From a market supply and demand perspective, the aggregate supply curve shifts leftward, output decreases relative to the original equilibrium point, and the prices rise.

The rise in industry monopoly fosters the formation of a competitive structure dominated by a few oligopolistic enterprises. The relationship between increased debt, aimed at expanding production capacity and profit, and the bankruptcy risk within an oligopolistic framework warrants in-depth investigation. Compared with the vicious competition among numerous small enterprises, monopoly or oligopolistic markets benefit from economies of scale. This structure reduces disorderly competition and resource wastage while promoting technological innovation within the industry. Hou and Kewei (2006) [51] explored the advantages of industry clustering in mitigating bankruptcy risk, emphasizing the role of entry barriers and reduction of creative destruction. On the other hand, while increased industry monopoly helps eliminate outdated and excessive capacity, it raises a critical question: Does it mitigate industry risk or does it amplify systemic risks associated with "too big to fail"? Further validation is needed.

This study incorporates the Herfindahl-Hirschman Index (HHI) to measure industry concentration. The HHI is calculated using the following equation:

HHI=∑i=1k(xix)^2=∑i=1kSi^2 (14)

*x*_*i*_ denotes the scale of the *i*-th enterprise within the industry, measured here by sales revenue, while k represents the total number of enterprises in the industry. A higher HHI value indicates a greater degree of industry monopoly. Notably, the HHI assigns greater weight to enterprises with a large market share, making it particularly sensitive to the distribution of market share among leading enterprises.

As evidenced by the regression results in Table 4, the impact of interest rate on the bankruptcy risk of the industry is negatively correlated (Model 4), aligning with the short-term effect. However, in the long term, interest rates primarily exert an intermediary effect through the degree of industry monopoly. The effect of the interest rate on the industry monopoly index is negative(Model 1), confirming that low interest rates promote greater industry concentration, consistent with the findings of Ernest Liu et al. (2022) [41]. A comparison of Model 2 and Model 3 indicates that industries with strong monopolistic characteristics are more likely to experience increased concentration following a decline in interest rates. This aligns with the conclusions of numerous related studies [50]. From Model 1, Model 4, Model 5 and Model 6, it is evident that industry monopoly (HHI) serves as a mediator in the transmission mechanism between interest rates and the average bankruptcy risk in an industry, with the intermediary effect accounting for 30%. The heterogeneity analysis in Model 6-Model 10 further reveals that higher industry concentration reduces the risk of industry bankruptcy at the average level. This is likely because the construction industry as a whole suffers from excess capacity, necessitating reductions to mitigate industrial risk(as monopoly markets typically produce less production than competitive markets). Notably, sectors such as coal, steel, cement, glass, and other building materials, alongside the construction and real estate industries, have long suffered from overcapacity. In contrast, for sectors like the decoration industry, where overcapacity is less pronounced, an increase in industry concentration may elevate risks.

**Table 4:** Interest rate, industry bankruptcy risk, and aggregation degree.

	(1) All	(2) HHI>0.07	(3) HHI <0.07	(4) All	(5) All	(6) All	(7) Materials	(8) Estate	(9) Build	(10) Decoration
VARIABLES	HHI	HHI	HHI	avDD	avDD	avDD	avDD	avDD	avDD	avDD
InterestRate	-0.0048***	-0.00793 ***	-0.00583 ***	-0.154 ***	-0.109 ***					
HHI					13.73 ***	14.21 ***	4.639***	13.24***	10.75***	-11.38***
PM	0.00028***	0.000294 ***	0.000375 ***							
HouseSale	-0.0075***	0.00220	-0.0111 ***	1.695 ***	1.788***	1.739 ***	2.115***	1.939***	0.850***	2.673***
avLoanR	-0.0074***	0.00456 **	0.00558 ***	-0.368 ***	-0.235**	-0.200**	-1.572***	1.178***	0.928***	-5.285***
avSCF	0.00622***	0.00934 ***	-0.0410 ***	-0.111 ***	-0.153 ***	-0.135***	0.662***	-1.437 ***	-2.536 ***	0.276***
LnGDP	-0.0048***	-0.0335 ***	-0.0118 ***	-1.830 ***	-2.038 ***	-2.044***	1.378***	-2.764 ***	-1.613 ***	-2.469***
Constant	0.136 ***	0.533 ***	0.178 ***	25.35 ***	26.41***	26.13 ***	-10.74***	35.11***	20.12***	38.12***
R-squared	0.223	0.183	0.678	0.338	0.358	0.356	0.231	0.669	0.637	0.409
Sample	283	283	162	283	283	283	56	120	76	31

**Note(s):** Standard errors in parentheses.

*** p<0.01

** p<0.05

* p<0.1

## 4. Robustness testing

This study employs the CCA method to estimate enterprise bankruptcy risk. Following the methodology of prior studies, liabilities are defined as the sum of current liabilities and half of non-current liabilities. Kealhofer et al. (1998) [52] analyzed numerous cases of defaulting companies, concluding that the default point should be equal to short-term liabilities plus half of long-term liabilities. However, this coefficient might not be appropriate for contemporary Chinese enterprises. Chi et al. (2012) [53], for instance, adjusted the coefficient using the credit spread of corporate bonds, finding that the coefficient for long-term liabilities of Chinese listed banks is approximately 0.7654. Additionally, Gilson et al. (1990) [54] observed that nearly half of the financially distressed enterprises avoided being liquidated through debt restructuring. This implies that enterprises may not go bankrupt immediately upon their asset market value reaching a critical threshold. Instead, liquidation bankruptcy occurs only when assets fall below a specific threshold. Consequently, the selection of the long-term liabilities coefficient may have a significant influence on the measurement of bankruptcy risk. Accordingly, robustness testing is conducted using three distinct coefficient settings: 0.2, 0.7, and 1 (represented by suffixes 02, 07, and 1 in the corresponding dependent variables in Table 5). The testing results (presented in Table 5) indicate that the selection of the non-current liabilities coefficient affects the regression coefficients to some extent. However, no substantial changes are observed in the sign, magnitude, or significance level of coefficients. Therefore, the robustness of our findings is verified, and conclusions remain unchanged.

**Table 5:** Robustness testing of different coefficients for non-current liabilities conversion.

	(1)	(2)	(3)	(4)	(5)	(6)	(7)	(8)	(9)	(10)	(11)	(12)
VARIABLES	DD02	DD07	DD1	DD02	DD07	DD1	avDD02	avDD07	avDD1	avDD02	avDD07	avDD1
InterestRate	-0.0612 ***	-0.045 **	-0.0431**				-0.147***	-0.142***	-0.131 ***	-0.097***	-0.093***	-0.085 ***
LoanR	-0.00335***	-0.00244**	-0.0023**									
HouseSale	1.447***	1.187***	1.094 ***	1.638 ***	1.263 ***	1.157 ***	1.389***	1.200***	1.060***	1.493***	1.301***	1.154***
LnGDP	1.550***	1.287 ***	1.245 ***	1.596 ***	1.231 ***	1.212 ***	-1.779***	-1.571***	-1.491 ***	-2.014***	-1.797***	-1.702 ***
LnAsset	-2.428 ***	-2.168***	-2.094 ***	-2.163***	-1.841 ***	-1.787 ***						
NPM	0.0227 ***	0.0204***	0.0198 ***	0.0287***	0.0268 ***	0.0265***						
SCF				-0.704***	-0.586 ***	-0.563 ***						
KZs				-0.117**	-0.104 **	-0.0874**						
avLoanR							-0.520***	-0.445***	-0.462 ***	-0.371***	-0.301***	-0.328 ***
avSCF							-0.0207	-0.096***	-0.0888***	-0.0687**	-0.142***	-0.132 ***
HHI										15.45***	14.88***	13.93***
Constant	18.42***	17.55 ***	16.86 ***	13.93 ***	13.41 ***	12.75 ***	24.88***	21.97***	20.82***	26.07***	23.11***	21.89***
R-squared	0.247	0.269	0.272	0.224	0.239	0.243	0.326	0.323	0.319	0.351	0.354	0.349
Sample	269	269	269	267	267	267	283	283	283	283	283	283

**Note(s):** Standard errors in parentheses.

*** p<0.01

** p<0.05

* p<0.1

## 5. Conclusion and policy implications

**Fig 15:**
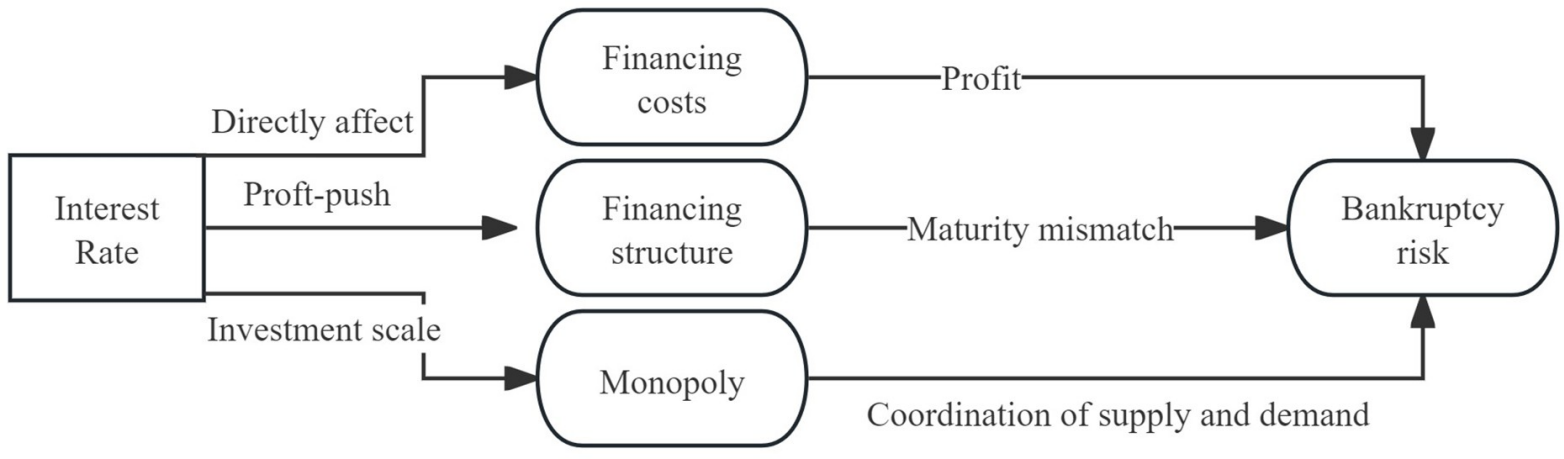
The transmission mechanism of interest rate to the bankruptcy risk.

This study utilizes data from the Chinese construction industry to investigate the effects of market interest rates on corporate bankruptcy risks, as well as the transmission mechanisms underlying these effects (illustrated in Fig 15). The findings indicate that interest rates are negatively correlated with bankruptcy risks across the construction industry. However, understanding the transmission mechanisms of these risks needs to consider not only traditional credit channels but also the long-term and short-term effects of interest rate fluctuations. In practice, higher interest rates can elevate financing costs, thereby influencing operating profits. However, the direct impact on bankruptcy risks is limited, and more risks arise from firms being compelled to adopt shortsighted strategies that sacrifice financial safety in the pursuit of profit maximization. Specifically, rising market interest rates prompt companies to increasingly rely on supply chain financing as a short-term substitute for credit financing. This reliance creates a mismatch between investment and financing terms, thereby heightening bankruptcy risks. However, over the long term, interest rate trends affect a firm’s production scale, which subsequently influences industry agglomeration. This dynamic affects bankruptcy risks through changes in the balance of industry output and sales. Notably, lower interest rates facilitate industry agglomeration, and increased monopolization within the industry tends to mitigate bankruptcy risks on average. This outcome is particularly relevant in the context of the construction industry’s persistent overcapacity. In essence, greater industry monopolization helps alleviate risks associated with overcapacity.

Given the significant impact of interest rates on critical industries in the national economy, monetary regulators should closely monitor industry-level risks and implement differentiated interest rate policies to proactively mitigate these risks and foster the sustainable development of industries. Specifically, two key considerations should be addressed:

Firstly, attention should be given to short-term interest rate risks to prevent the formation of speculative credit bubbles. Fluctuations in interest rates compel firms to adjust their financing structures in the short term, which may result in excessive reliance on supply chain financing. This mismatch between "short-term borrowing and long-term investment" creates increased bankruptcy risks for individual firms and amplifies the interdependence of risks across the industry chain, thereby magnifying overall industry risk. Therefore, regulators should monitor the short-term interest rate risk capacity of individual sub-industries within the construction sector and establish targeted benchmark interest rates to guide the credit market, thus preventing the accumulation of commercial credit risks.

Secondly, long-term monetary policies should focus on optimizing the industrial structure. Changes in long-term interest rates can influence industry agglomeration, which in turn affects industrial-level risk. The intermediation effect of industry agglomeration can help balance supply and demand within the sector. Therefore, when formulating long-term monetary policies, it is essential to integrate industrial policy considerations and align them with future market development trends.

## Supporting information

S1 Data (ZIP)
